# Mapping the health systems response to violence against women: key learnings from five LMIC settings (2015–2020)

**DOI:** 10.1186/s12905-021-01499-8

**Published:** 2021-10-10

**Authors:** Shegufta Shefa Sikder, Rakhi Ghoshal, Padma Bhate-Deosthali, Chandni Jaishwal, Nobhojit Roy

**Affiliations:** 1 CARE USA, 151 Ellis St NE, Atlanta, GA 30303 USA; 2 CARE India, No.14, Patliputra Colony, Patna, Bihar 800013 India; 3 WHO Collaborating Centre for Research in Surgical Care Delivery in LMICs, Mumbai, India; 4 Independent Consultant, Mumbai, India; 5 Rollins School of Public Health, Emory University, 1518 Clifton Rd, NE, Atlanta, GA 30322 USA; 6 Department of Global Public Health, Karolinska Institutet, 171 77 Stockholm, Sweden; 7 The George Institute for Global Health, New Delhi, India

**Keywords:** Health system response, Violence against women, Gender-based violence, One-stop centers, Low- and middle-income countries

## Abstract

**Background:**

Violence against women (VAW) is a global challenge, and the health sector is a key entry point for survivors to receive care. The World Health Organization adopted an earlier framework for health systems response to survivors. However, documentation on the programmatic rollout of health system response to violence against women is lacking in low and middle-income countries. This paper studies the programmatic roll out of the health systems response across select five low- and middle-income countries (LMIC) and identifies key learnings.

**Methods:**

We selected five LMIC settings with recent or active programming on national-level health system response to VAW from 2015 to 2020. We synthesized publicly available data and program reports according to the components of the WHO Health Systems Framework. The countries selected are Bangladesh, Brazil, Nepal, Rwanda, and Sri Lanka.

**Results:**

One-stop centers were found to be the dominant model of care located in hospitals in four countries. Each setting has implemented in-service training as key to addressing provider knowledge, attitudes and practice; however, significant gaps remain in addressing frequent staff turnover, provision of training at scale, and documentation of the impact of training. The health system protocols for VAW address sexual violence but do not uniformly include clinical and health policy responses for emotional or economic violence. Providing privacy to survivors within health facilities was a universal challenge.

**Conclusion:**

Significant efforts have been made to address provider attitudes towards provision of care and to protocolize delivery of care to survivors, primarily through one-stop centers. Further improvements can be made in data collection on training impact on provider attitudes and practices, in provider identification of VAW survivors, and in prioritization of VAW within health system budgeting, staffing, and political priorities. Primary health facilities need to provide first-line support for survivors to avoid delays in response to all forms of VAW as well as for secondary prevention.

**Supplementary Information:**

The online version contains supplementary material available at 10.1186/s12905-021-01499-8.

## Background

Globally one in three women experiences violence in her lifetime [1]. For many women the violence is perpetrated by an intimate partner or family member [2]. The prevalence of reported intimate partner violence ranges from 15 percent in Japan to 71 percent in Ethiopia [1, 2]. Such violence has far-ranging reproductive, sexual, and mental health consequences [3]. Survivors access health facilities for medical treatment and care of the symptoms and injuries [4]. Thus, health providers are uniquely positioned to provide support to survivors, beyond the immediate medical care [5]. However, support beyond the initial clinical response has not been successfully integrated into health care systems [6].

In 2014, the World Health Assembly stated that the health system is a key entry point for survivors to receive help and discussed a commitment for a health response as well as a framework for country strategies [7]. In 2015, the World Health Organization (WHO) adopted an earlier framework for health systems response to survivors [8]. This framework comprises the key elements within the domains of service delivery, leadership and governance, information, health infrastructure, health workforce development, coordination, and financing for health systems response to violence against women (VAW) [8]. In this paper, we focus on VAW because women and girls encounter the majority of violence directed at people based on their sex or gender identity [5, 7]. The term VAW includes any physical, sexual, emotional, or economic violence directed at women and girls by an intimate partner, family member, acquaintance, or stranger [4, 7]. Health systems have a critical role in addressing VAW within a multi-sectoral approach [5].

Despite the framework on health systems response to VAW, limitations remain in implementation. Typical challenges survivors face at health facilities include insensitive approach of providers, re-traumatization of her experience of violence, and victim-blaming [9, 10]. Consequently, many survivors are forced to return to the place of abuse without any safety plan or support in place [11]. Cultural beliefs normalizing VAW reinforce the lack of support for survivors and exacerbate the challenges of proper health care delivery to survivors. Other challenges to providing a health system response include inadequate infrastructure and lack of coordination between the health sector and other sectors [12–14]. Several governments have begun to focus on putting in place structural support systems for survivors. Health systems interventions include implementation of appropriate policies and guidelines, focused budgeting, standardising protocols, and integration of a gender-sensitive response at the facility level [5]. In addition, in 2013 the WHO released its Policy and Clinical Guidelines for Response to Intimate Partner Violence. However, they emphasized the gaps in evidence from low- and middle-income countries (LMICs) as most of the evidence was based on systematic reviews of evidence from largely high-income settings [15]. The 2015 Lancet Series on VAW discussed the evidence base on addressing VAW and the role of the health sector, highlighting the need for evidence on addressing VAW in LMICs [5, 6].

Against the context of high burden of VAW along with challenges to addressing health system response to VAW in LMICs, this paper seeks to identify programmatic learnings from recent implementation of national-level health systems response to VAW across five selected LMIC settings between 2015 and 2020.

## Methods

We purposively selected LMIC countries that had recent or active national-level health system programming for response to VAW between 2015 to 2020 and where we had access to practitioners directly engaged in national rollout of these responses, specifically Bangladesh, Brazil, Nepal, Rwanda, and Sri Lanka. To understand the responses and programs, we included all published and unpublished documents that detailed the rollout of national level health system response to VAW in these five LMIC settings. We categorised the information by the components of according to the Health Systems Framework for Response to VAW in the specified time period.

Two authors (SS and CJ) applied keyword search on Google Scholar (keywords included “health system programming for response to violence against women”) specific to the five selected countries and time frame (2015–2020). SS searched for articles in two settings (Bangladesh and Brazil) and CJ applied searches for three settings (Nepal, Sri Lanka, and Rwanda), with each individually recording whether articles matched inclusion criteria. Through mutual agreement, SS and CJ identified 46 articles, 35 of which were excluded due to time frame (outside of the 2015 to 2020 time), geography (not occurring in the five selected settings), or scope of programming (not inclusive of health system response to VAW). This keyword search yielded 11 relevant publications [17–27], including two articles SS and CJ agreed to be relevant to LMIC programming for health system response to VAW more broadly despite not specifying the selected five countries [18, 42]. Then, SS and CJ together also consulted program practitioners (listed in Acknowledgements) involved in national programmatic rollout in these five settings and requested them to identify program reports or government protocols on health system response to VAW that may have been missed. The program practitioners identified a total of 26 documents, 19 of which fit the relevant time frame (2015 to 2020) and which we included in our analysis [28–46].

The 11 publications from the keyword search combined with the 19 documents identified through consultation with program practitioners yielded a total of 30 documents [17–46] which described national health systems response to VAW in these five settings between 2015–2020. To guide our analysis, we used the Health Systems Framework as our analytic framework to address VAW to identify key themes across the seven key components of the framework: (1) service delivery, (2) health infrastructure, (3) health workforce development, (4) leadership and governance, (5) financing, (6) health information systems, and (7) coordination and community engagement [8]. CJ was responsible for initial identification and categorization of information from the identified articles that corresponded to particular components of the framework. SS was responsible for synthesis of information across components through discussion with all co-authors [16].

## Results

### Key summaries

Table 1 summarizes the key findings from the five settings—Bangladesh, Brazil, Nepal, Rwanda, and Sri Lanka. While all five LMIC settings have implemented in-service training as key to addressing provider knowledge, attitudes and practice, significant gaps remain in addressing frequent staff turnover, provision of training at scale, and documentation on the impact of training. Health system protocols for VAW address sexual violence but do not uniformly include clinical and health policy responses for other forms of violence, such as emotional or economic violence. Providing privacy to survivors within health facilities remains a universal challenge. Nepal’s health system response to VAW represents the most comprehensive rollout across key components. Protocols exist for providing a health systems response which includes 24-h operationalization of One Stop Centres (OSCs; which are called One-Stop Crisis Management Centers or OCMCs in Nepal), appointment of designated staff, provider sensitization and training on clinical response. Further, the VAW data is centrally recorded and managed through the Health Management Information System (HMIS). Community engagement for identification of survivors and referral to support services is a prominent part of Nepal's health systems response [20, 29, 31, 34, 35]. [OSCs provide multi-sectoral case management for survivors, including health, welfare, counselling, and legal services in one location and will be described further in the following section.]

**Table 1:** Key summaries from health system response to VAW across five selected LMIC settings

Bangladesh	Brazil
Bangladesh has OSCs at regional and district levels for responding to VAW. NGOs in Bangladesh have implemented screening checklists and referral flow-chart to facilitate identification of VAW and provision of services at sub-district health clinics. Collaboration between the Directorate General of Health, and the Ministry of Women and Children’s Affairs strengthen the government’s commitment to respond to VAW. Two significant challenges are proper infrastructure and ensuring privacy within the facility setting. A bulk of the financing for health system response is provided by international donors	In Brazil, much of the health system response to VAW is focused on sexual violence. While Brazil does not have OSCs, hospitals around the country provide response to sexual violence including medical services and psychosocial counseling. One of the most prominent laws, the *Maria de Penha law*, criminalizes domestic violence. Health care providers are mandated to report any incident of VAW, though many are untrained and have unsupportive attitudes towards VAW. Infrastructure challenges abound, compromising survivor identification and provision of survivor-centered care

Nepal	Rwanda
Nepal has OSCs in all districts, offering a full range of support services, with designated trained staff nurses. Service delivery at OSCs is protocolized. The Prime Minister’s Office monitors VAW data. Allocation of 15 percent of funds for awareness and response to VAW at community level underlines the government’s priorities. Assuring client privacy in health settings is a challenge. Provider training and evaluation takes place on an ongoing basis	Rwanda has policies for health systems response to VAW, and its OSC model exists across most districts. The government receives substantial financial support from foreign donors even as its own allocation is weak. Despite ranking high on global gender indices, societal attitudes indicate widespread normalization of VAW. Challenges in this setting include lacking infrastructure which compromise survivor privacy, weak intersectoral coordination, and poor monitoring and documentation of implementation

Sri Lanka	
Sri Lanka provides OSC services in all districts. The cadre of Public Health Midwives focus on empowering survivors. There are laws on health system response to VAW; the government has demonstrated increasing responsibility for financing health sector response to VAW. A nodal person is designated within the health system who has the authority to work across ministries on strengthening the multisectoral VAW response. Gaps include lack of documentation of VAW cases and poor monitoring of implementation

1. Models of service delivery

Service delivery for VAW includes psychosocial support, medical treatment, multi-sectoral referral including within the health systems [15]. As seen across these five settings, health system protocols for VAW specify medical services and supplies for response to sexual violence but do not uniformly include clinical and health policy responses for other forms of violence (such as emotional or economic violence) [31–33, 37, 45].

In Bangladesh, nongovernmental organization (NGO) clinics implemented a routine enquiry pilot for identification of violence among women seeking health services. The NGO Health Service Delivery Project (NHSDP) was a health service delivery project in Bangladesh funded by the United States Agency for International Development. This project supported the delivery of reproductive, maternal, and child health services through a network of rural and urban local NGO clinics that targeted poor and underserved women of reproductive age [28]. The NHSDP trained its staff on VAW including aspects of gender and women-centered care, implemented a routine enquiry pilot, and developed a national protocol on health response to VAW [28].

In Rwanda, the Maternal and Child Survival Project applied quality assurance standards for VAW at OSCs nationally, reaching over 1500 clinicians [30]. This work included routine enquiry for VAW integrated into family planning and antenatal counselling in 12 health facilities. Provider training did not include gender sensitization and key aspects of women-centered care [30]. Staff reported that disclosure rates of violence were extremely low (one to two percent in the first six months) of this routine enquiry pilot [30]. Providers reported feeling overstretched and overburdened, while client feedback indicated that providers were insensitive when inquiring about violence. These challenges precluded the expansion of the routine enquiry intervention.

One-Stop Centers (OSCs) were found to be the most dominant model of care implemented by the health systems in four countries (Bangladesh Nepal, Sri Lanka, and Rwanda). An OSC provides multi-sectoral case management for survivors, including health, welfare, counselling, and legal services under one roof with the intention of minimizing referrals and the need to have survivors repeat their accounts [18]. In Bangladesh [33], Nepal [29], Rwanda [37] and Sri Lanka [32], OSCs are located within hospitals. While Brazil does not have OSCs, hospitals around the country provide response to sexual violence including medical services and psychosocial counseling [38, 46]. While Rwanda and Sri Lanka’s OSCs were set up by the health system, the OSCs in Bangladesh and Nepal are set up as large multi-sectoral programmes with delineated health sector roles and responsibilities [26, 29, 33, 37]. Outside of OSCs, in all five countries, hospitals in the health sector include response to sexual violence through medical services, psychosocial counseling and referral as outlined in their respective health systems protocols [24, 31–33, 37, 45]. A summary of the models of care in each setting is shown in Table 2. Available details on client volume and services at OSCs in each setting are shown in Additional file 1.

**Table 2:** Summary of models of health services for VAW by setting

Country	Health system organization	Models of health service delivery for VAW	Coverage of health services for VAW
Bangladesh	Bangladesh has a pluralistic unregulated healthcare system, with government, for-profit private sector, not-for-profit private sector (mainly the nongovernmental organizations), and the international development organizations as actors [48]	The Multi-Sectoral Programme on VAW is led by the Ministry of Women and Children’s Affairs and includes coordination for OSCs, one stop cells, and regional trauma centers. The Ministry of Health and Family Welfare is responsible for ensuring health service provision for VAW response services across hospitals and OSCs [33]	Bangladesh has OSC Cells at 47 out of 62 district hospitals and in 20 of the largest sub-district health complexes which provide information on services available for VAW including health care, police assistance, legal advice, psychosocial counseling, rehabilitation activities, and reintegration programmes [33]
Brazil	Universal and free public health care is provided to all Brazilians as a constitutional right within the Unified Health System [49]	Hospitals around the country provide response to sexual violence including medical services and psychosocial counseling [46]. In Brazil, a violence prevention nucleus is responsible for multi-sectoral coordination, training, and surveillance of domestic violence cases at primary health clinics [38]	Since 2017, seven women’s houses have been established across regions of Brazil. There are no centers at local levels [38]. Approximately 60 hospitals around the country with programs respond to sexual violence [46]
Nepal	Health care services in Nepal are provided by both public and private sectors. While universal health coverage policy was launched in 2015, it is yet to be widely implemented [50]	Nepal has a model of hospital-based OSCs, with training and quality control provided by the Government in collaboration and support from Jhpiego (between 2015 and 2020) [35]	In 2020, each of Nepal’s 77 districts reported having an OSC [29]
Rwanda	Rwanda follows a universal health care model, through a community-based health insurance scheme [51]	Rwanda’s OSCs (known locally as *Isange*, or “to feel at home) are centers located within hospitals [37]	Rwanda has OSCs functional in 21 of its 25 districts staffed by physicians, nurses, social workers, psychiatrists and police [37]
Sri Lanka	Sri Lanka has a universal health care system through public (general) hospitals and free outpatient departments [52]	Within Sri Lanka, a network of centers is usually placed within the outpatient departments of hospitals. The centers are called *Mithuru Piyasa* (“friendly haven”) established in 2008. *Mithuru Piyasa* centres are staffed by a medical officer and a nursing officer in outpatient and emergency care settings within hospitals between 8 am and 5 pm. With a shortage of staff, the centres do not provide for 24/7 provision of services [32]	By the end of 2016, 21 out of 26 districts had a district-level hospital with a *Mithuru Piyasa* [32]

In all five settings, staff at hospitals are designated and trained in protocols detailing response to VAW [25, 31–33, 37]. Referrals to OSCs come largely from police and directly from communities (Additional file 1). Health system protocols for VAW specify that hospital departments, especially emergency and specialized departments, should refer clients for additional medical services [25, 31–33, 37]. Gaps remain in training of health providers in identification of signs and symptoms of VAW [23, 29, 32, 33, 37].

In Brazil, protocols include a team approach implemented to responding to VAW, in which all medical cadres are deemed responsible for clinical response, identification, treatment, and referral [23]. A violence prevention nucleus consisting of a social worker, psychologist, and nursing technician is responsible for multi-sectoral coordination, training, and surveillance of domestic violence cases at primary health clinics [38]. In Nepal and Sri Lanka, responsibilities of health care providers include identification, confidentiality, and obtaining informed consent, providing first aid and LIVES,^1^ history taking, medical management, medico legal services, referral, and follow up [31, 32]. In Sri Lanka, the public health midwife is tasked with identifying issues related to VAW and referring and connecting survivors to the health system. Specific to sexual violence, medical officers are identified as responsible for medical management and response [32].

While a survivor-centered approach remains central to the framework of Health Systems Response to VAW, reviews find that providers’ beliefs, values, and attitudes often restrict their provision of survivor-centered care [12, 18, 23]. In addition to insensitive provider attitudes towards VAW, the use of traumatic procedures from health care providers among survivors have been documented [54]. In Brazil, Nepal, and Sri Lanka, harmful practices such as virginity testing and assessment of hymen for sexual habituation remain within clinical protocols despite supportive policies on health system response to VAW [53].

A 2020 review of one-stop centers (OSCs) found that staff engaged in victim-blaming in most settings [18]. Though Brazil criminalizes domestic violence, many health providers are “still reluctant to engage with the issue [23].” Even staff within violence prevention nucleus felt unprepared to respond to VAW and were uncertain about their roles and referral pathways for survivors [38]. Data from health providers in Rwanda showed acceptance and normalization of VAW [40]. Across all the five settings, VAW, particularly violence by the intimate partner/husband is normalized in society, with 28 to 53 percent of women agreeing that wife-beating is justified for any reason across the five settings [54–60]. Health providers are part of the same society, and commonly share the same attitude towards acceptance of violence and of blaming the survivor for the violence [61, 62].

2. Health infrastructure

Providing privacy and confidentiality, availability of medicines and other supplies constitute infrastructure. In all the settings, data showed that providing privacy to the survivor emerged as a challenge. Additional challenges in all five settings included lack of furniture, information and communication material, instruments, and medicines, use of space and equipment designated for OSCs by other departments, and excessive patient load leading to overcrowding [17, 18, 25, 26, 44]. In Nepal, OSCs were ranked as providing better privacy and confidentiality than health facilities, and this was attributed to the protocolized nature of VAW response at the OSCs. A score card used to rank OSCs in Nepal showed 82 percent of assessed OSCs operating with adequate space and equipment or supplies [34, 35]. In all other settings, infrastructural challenges included lack of adequate health provider training besides the lack of space to ensure privacy [17, 18, 25, 26, 44].

3. Health workforce training/development

Stakeholder training is built into the standard operating procedures for health systems response to VAW in all settings [21, 31–33, 37]. Frequent staff turnover remains a challenge to capacity building on VAW response. Health provider training programs in Nepal focus on improving competency and skills; nurses are mandated to identify survivors and doctors to provide medical treatment and to report medico-legal cases [29]. Nepal addressed staffing gaps in its 2016 revised OSC Operational Guideline by appointing a minimum of one medical doctor and two to three staff nurses to the OSC for 24-h service availability [34]. A 2020 report on Nepal’s OSCs indicates that the majority addressed these staffing changes to be operational 24 h a day [29]. Following training on health systems response, providers noted a new sense of responsibility they felt to support the survivor get justice, while noting that doctors not being relieved from their regular duty if they have to attend a court hearing, remains a challenge [29]. The United Nations Population Fund (UNFPA) and NGO partners in Bangladesh support the government in providing staff training; they have developed a screening checklist, referral flow-chart, counselling guideline and provided training to the providers for essential services in their clinic networks [28]. Monthly review meetings with health providers, outreach workers, and their supervisors are conducted. The National Protocol for Health Sector Response to VAW in Bangladesh requires health providers to provide psychological first aid to survivors, medico legal examination and proper documentation [33]. Sri Lanka focuses on training and capacity building, both for health providers and the public health midwife. Sri Lanka introduced VAW as a module for public health midwives focused on knowledge-building, skill development for addressing and preventing VAW in the community, as well as on positive provider–client interaction and communication [32]. Since 2015 the government in Rwanda has focused on strengthening and scaling its OSC model in all provinces, improving documentation, implementing quality assurance standards, providing specialized support to children and adolescent survivors, training providers on first-line support for survivors and post-GBV health care, making appropriate referrals, and strengthening referral pathways, with help from its non-governmental partners [37].

In Sri Lanka, health providers including medical officers, nurses, midwives, and their supervisors are trained in response to VAW [32]. The medical officer for maternal and child health at the district level is tasked with coordination between district and divisional levels [32]. In Nepal the OSCs have designated staff for managing/responding to cases that come, and usually a nurse or paramedic and a psychosocial counsellor [34]. Information on the process of designating a nodal person for managing VAW cases from the other three settings is vague.

While all countries reported that they train in-service providers, there was no documentation on impact of training on provider knowledge, attitudes, or practices. In Bangladesh, in addition to initial training, the NGO Health Service Delivery Project integrated discussion on provider attitudes on VAW into monthly meetings between health staff and supervisors besides refresher trainings [28]. In Nepal, a 2016 study indicated a lack of training and knowledge around VAW among health providers [19]. While impact data on provider change in attitudes resulting from training on VAW were not available, staff nurses who received psychosocial counselling training noted how they have changed the language they use with survivors and have become aware of how to show sensitivity, respect, and empathy to survivors [29]. Nepal’s Ministry of Health and Population introduced an in-service for medical doctors on autopsy and clinical medico-legal training, although training has not been initiated in recent years. A pre- and post- intervention study of a training intervention for public health midwives on response to IPV in Sri Lanka found that role plays, field handbooks and cultural sensitivity training were important components for an effective training program [17]. However, impact data on provider change in attitudes was lacking.

While all countries reported that they train in-service providers, there was no documentation on impact of training on provider knowledge, attitudes, or practices. In Bangladesh, in addition to initial training, the NGO Health Service Delivery Project integrated discussion on provider attitudes on VAW into monthly meetings between health staff and supervisors besides refresher trainings [28]. In Nepal, a 2016 study indicated a lack of training and knowledge around VAW among health providers [19]. While impact data on provider change in attitudes resulting from training on VAW were not available, staff nurses who received psychosocial counselling training noted how they have changed the language they use with survivors and have become aware of how to show sensitivity, respect, and empathy to survivors [29]. Nepal’s Ministry of Health and Population introduced an in-service for medical doctors on autopsy and clinical medico-legal training, although training has not been initiated in recent years. A pre- and post- intervention study of a training intervention for public health midwives on response to IPV in Sri Lanka found that role plays, field handbooks and cultural sensitivity training were important components for an effective training program [17]. However, impact data on provider change in attitudes was lacking.

4. Leadership and governance

Government prioritization of resources and interventions is essential to changing practices. All settings have included VAW in their health policy and programmes. Responding to VAW became a health policy issue in Nepal in 2010, and the government developed a National Plan of Action against VAW that built in a health sector response. The National Plan foregrounded the need for building a multi sectoral health response for GBV [19]. The Ministry of Health plays a central role in executing this Plan and has standard operating procedures for OSCs detailing the management of the centres, including infrastructure and resources, and the pathways for rolling out a multi sectoral coordination [34]. In Sri Lanka, the Family Health Bureau under the Ministry of Health is responsible for all VAW services that are provided by the state health system [32]. The National Policy on Maternal and Child Health describes the approaches and support for prevention and management of VAW and mandates capacity-building for health providers [26] as well as comprehensive health-sector based response in OSCs [22]. Bangladesh has developed a comprehensive protocol for the Women Friendly Hospitals Initiative which centrally acknowledges VAW as a health concern and developed a National Action Plan on Violence against Women 2013–2025 which outlines multisectoral coordination [41, 43]. The multisectoral program on VAW is jointly implemented by the governments of Bangladesh and of Denmark and led by the Ministry of Women and Child Affairs, Bangladesh.

Rwanda criminalized VAW in 2008, and the Ministry of Gender and Family Promotion developed National Strategic Plan for Fighting Against Gender-based Violence (2011–2016) [44]. Rwanda has a Gender Monitoring Office which has a mandate of monitoring compliance to gender equality principles [40]. From 2003 onwards, Brazil rolled out gender based public policies to manage women victims of VAW in healthcare settings, highlighting the primary health care (PHC) system as an important context [21]. In 2006 the country implemented the *Maria da Penha* Law which marks out domestic violence as a crime; the next year Brazil launched the first national policy on VAW, delineating the role and goals for the health sector [27]. Other policies include the National Special Secretary for Women's Policies (responsible for developing intersectoral action plans focused on women, targeting particularly health, security, education and reduction of violence), the National Policy to Combat VAW, and the National Policy for Humanization in Health (focused on humanization of all health care approaches). These policies have been implemented into primary health care settings through the Family Health Strategy [21].

5. Financing

Fund allocation is required for service training of providers for VAW response, for appointment of trained staff for running OSCs and providing other services for health system response, and necessary infrastructure [5]. The funds for OSCs come from government ministries of health in Nepal, Sri Lanka, and Rwanda [26, 34, 44]. In Bangladesh, the majority of funding for OSCs comes from international donors (including the Danish government for the Multi-Sectoral Programme on VAW) [33]. In Sri Lanka the OSC program is financed by the Ministry of Health, while international donors are only in a supportive role [26]. The OSCs in Rwanda were initially co-funded by the United Nations and the Government of Rwanda [44]. Local-level prioritization is well placed in Nepal, where each Village Development Committee allocates 15 percent of its budget for women’s issues, with microplanning occurring through Health Facility Management Committees. Funds are available to reimburse the healthcare and legal expenses incurred by survivors, even though complex regulations, and administrative hoops challenge access [34]. Recent changes in the federal system in Nepal leave uneven funding of multi-sectoral services for VAW survivors, such as safe homes and rehabilitation services [34, 35]. In Brazil, there is a national pact, with rules of fund transference between national government and the states, but the budget for VAW was hugely cut recently [25, 63].

6. Health information system

Documentation and information management in Nepal includes VAW data recorded at the national level on two different platforms, from all health facilities through the Health Management Information System (HMIS), and from the OSCs routed to the Prime Minister’s Office [35]. The VAW data is disaggregated along sex, age, type of violence, caste, ethnicity and disability [35]. Additional indicators note the services provided to survivors and referrals made. Rwanda and Nepal are the only countries in this study which report data on VAW through HMIS. Since 2012, Rwanda has worked on streamlining its quality of health data which is collected from the community health workers and health facilities to HMIS and has integrated facility-level as well as household-level data [30]. While the Multi-Sectoral Programme on VAW in Bangladesh indicates plans for a nationalized database on VAW, such a database does not exist. The Bangladesh Bureau of Statistics collects household level survey data on VAW once every four years [33]. From the national VAW hotline, non-identifiable data is accumulated on volume of calls, types of VAW experienced, and referrals [33]. In Brazil, reporting on VAW is the responsibility of state and local authorities with data accumulated at sub-national levels [24]. Sri Lanka does not capture data on medico-legal cases nor reports through HMIS [26].

7. Coordination and community engagement

Coordination to facilitate internal and other sectoral referrals and community engagement are important axes of the Health Systems Framework to Address VAW. OSCs in Nepal have protocolized multisectoral coordination such as with the legal, judicial, and livelihoods. The referral pattern of OSC survivors shows community awareness about services as a portion of survivors reach OSCs directly [29]. In Sri Lanka’s 2016 Demographic and Health Survey, 13 percent of surveyed women were aware of *Mithuru Piyasa* services [59]. In Sri Lanka since these centers are located within the hospital, intra-facility referrals are relatively smooth, though other-sectoral referrals are not formalized. Recognising the importance of engaging the community, Sri Lanka prioritized the use of information education communication material, with public health midwives responsible for community engagement. Given their access to the community, midwives remain critical for identifying survivors, especially those women who otherwise would continue to bear the violence in silence [17].

In the rest of the settings, structured intersectoral coordination and referral are lacking. Changes within the federal system in Nepal have resulted in uneven funding of multi-sectoral services for VAW survivors, such as safe homes and rehabilitation services [35]. The NGO Jhpiego oriented select community health volunteers in Nepal for identification and referral of survivors, showing that with appropriate training and safety mechanisms, community health workers can raise community awareness about VAW, facilitate support for survivors, and help prevent harmful practices [20]. In Bangladesh, while the Multi-Sectoral Programme on VAW does not have an explicit community engagement component, the initiative runs TV ads and radio spots for public awareness on the availability of VAW services [33]. The world’s largest NGO (Building Resource Across Communities, or BRAC), as well as other NGOs, have implemented programs focusing on improving community engagement and awareness of VAW, though these services are not directly linked to the Multi-Sectoral Programme on VAW [36]. In Brazil, networks of psychologists and social workers, activists, and women-led organizations increase awareness on VAW, though efforts are not formally linked to available health services [24]. Rwanda’s Protocol on Treatment of Survivors of VAW demonstrates a model of inter-sectoral coordination for VAW and includes provisions for safety planning and community resources for survivors, though formal coordination with other sectors for VAW support is not protocolized [37].

## Discussion

VAW is a health care issue requiring a health system response. While evidence on the effectiveness of the current responses is limited, key learnings are evident by component of health system response. All the five countries have integrated response to sexual violence, which is important especially in low-resource settings where health care providers are likely to encounter clients experiencing high levels of violence [5]. However, documentation on response other forms of VAW such as emotional or economic violence was lacking in every setting. Recognition of VAW in health policy and programs is a crucial prerequisite in rolling out a health system response. Health policies targeted to address VAW and political intention translated into allocation of funds, training of providers and setting up of services within health facilities in the five settings. As in Brazil, the change in political leadership which is not supportive of gender issues has adversely affected the programming on VAW [63]. A well-funded system is critical to ensure women-centred response, and the source of funding in these countries varied from it being part of the government’s health budget (Sri Lanka) to the budget of Ministries of Women and Children to donor agencies. An incomplete prioritization by the government can limit the sustainability of the program as well as the full integration of VAW response within the health system.

WHO’s Clinical Guidelines on Response to Intimate Partner Violence do not recommend a single model for service delivery, since women are likely to enter health facilities to access services through various departments such as antenatal care, family planning, obstetrics and gynecology, medicine, paediatrics and/or psychiatry [15]. Emergency departments are more likely to receive cases of physical assaults, injury, or rape. It is therefore critical that VAW response is integrated across these services and not provided as a silo. Four of the five countries studied focused VAW service provisioning through OSCs with limited integration of VAW response across hospital departments. While OSCs are centered at tertiary or secondary levels of care, initiatives such as Healthcare Responding to Violence and Abuse (HERA) underscore the need for primary health care services for VAW prior to referral to higher-level health facilities [64]. In Brazil, Nepal, Sri Lanka, and the occupied Palestinian territories, the HERA initiative has already reported on readiness of primary care systems to respond to violence against women and will evaluate approaches to build clinician capacity for response and referral for VAW at the primary care level, with results to be completed in 2021 [64, 65].

The inherent advantages and disadvantages of a OSC model define the organisation of these services. While these were set up in district and tertiary hospitals, they were funded by the Ministry of Women and Children’s Development or external donors which limited their integration into the hospital system [18]. The referral pathways, where available, indicate that most of the survivors coming to the OSCs were brought in by the police or referred by civil society organizations, particularly for survivors of rape, assaults, and burns. The OSCs therefore have ensured that all women and girls reporting with rape or assault are being provided a standard of care.

In Bangladesh, Nepal, Rwanda, and Sri Lanka, OSCs are located within the health facility, which facilitates medical support for survivors [38]. Survivors seeking VAW services at OSCs receive a standard of care according to protocols. Also, OSCs in all settings were able to ensure better privacy than the facilities in corresponding health settings [18]. While OSCs have resulted in provision of care for VAW at tertiary and district levels, OSC evaluations call for an adapted model of OSCs, in which tertiary and regional hospitals have specialized OSC units for comprehensive VAW response services, and lower-level primary and district facilities provide first-line support with referral for specialized services as needed [33].

The WHO recommends system-level changes to include standard operating procedures, referral linkages, building strong leadership, supportive supervision and availability of adequate infrastructure for ensuring privacy and confidentiality [7, 15, 47]. Training is essential but not sufficient on its own and has to be supported through other changes in the system which affect clinical practice. System-level changes such as protocols and information education communication material were introduced in most settings. However, there was no mention of any mechanism for monitoring the implementation of these, or any provision for responding to any violation or non-adherence. Recruitment and retention of staff emerged as an issue across every setting. In Rwanda, practitioners suggested staggering staff rotations in the future to prevent service stoppages for VAW response, particularly for forensic evidence collection [30].

While each setting has acknowledged ongoing training as key to changing attitudes and building health systems response, all settings focused on in-service training without including VAW in pre-service curriculum. Within in-service training, challenges include lack of availability of staff and frequent turnover. As conducted in Bangladesh, regular review meetings included discussion on VAW response with health providers, outreach workers, and their supervisors for ongoing prioritization of health response [28]. Short, targeted, in-service, simulation-based learning activities, spaced over time and reinforced with structured, ongoing practice sessions (known as low-dose, high-frequency trainings) were found to be less time-intensive than traditional in-service trainings and resulted in provider retention of key content [66, 67]. The structure in Sri Lanka whereby of all providers trained in VAW, one is designated nodal and is given additional training, is worthy of mention, reflecting the investment of the government in the issue [22, 32]. Further, to have the person work across ministries is a progressive move, since this would facilitate collaboration and coordination across ministries, and thus sectors.

Studies from other LMICs such as Pakistan, Serbia, Palestine, have also reported a lack of sensitivity to VAW, victim blaming attitudes, lack of awareness about available services [68–70]. Colombini et al. report on several barriers and challenges in conducting training of health care providers in LMICs such as their lack of time, heavy patient load/case load, high staff turnover, infrastructural issues amongst others [9]. These are compounded by lack of gender sensitivity in medical education, archaic and unscientific forensic medical practices such as virginity testing and two-finger test, and lack of recognition of VAW as a health care issue due to biomedical approach [53, 71].

Responding to VAW needs a multi-sectoral approach and so the health system also needs to coordinate with other sectors as well as engage communities on the health consequences of VAW. Sri Lanka, Nepal and Bangladesh have paved the way for such coordination through engagement of midwives, female community health volunteers, and multimedia, respectively. Coordinated, multi-sectoral response would enable health systems to fulfill their role in preventing violence and caring for survivors.

## Strengths and limitations

This paper contributes to the research on health systems response to VAW identifying key learnings from LMIC contexts. These learnings may be useful for program planning of health system response to VAW in other similar settings. Given that the burden of VAW is significantly high in LMICs, the learnings would be valuable for policy makers and other stakeholders.

Limitations of this paper include purposive selection of the five settings and varying levels of information across settings. To offset these limitations, we presented information according to components of the Health System Framework for VAW response where it was available. Most of the evidence, particularly on OSCs, does not evaluate effectiveness of the OSC model, and thus it may be difficult to discuss lessons learned and limitations when many of these models have not been evaluated. As we focused on documents available between 2015 and 2020 in order to understand recent implementation lessons, we excluded earlier documents which were available from these settings.

## Conclusion

This paper provides compelling examples of how LMICs have operationalized health system response to VAW. Significant efforts have been made to protocolize delivery of care and support to survivors, primarily through one-stop centers. However, OSCs need to be linked to other services within the hospitals as well as primary health facilities. The training of health care providers in identification of abuse and provision of first-line psychological support needs urgent attention. The WHO’s Clinical Guidelines on Response to Intimate Partner Violence need to be operationalized for all forms of violence and not limited to sexual violence only through training, changes in clinical practice, documentation, coordination with other sector and communities. Additional research on accessibility, acceptability, and quality of OSC services is needed. Future research could focus on understanding effective strategies to improve health provider attitudes and clinical practices specific to all forms of VAW to advance health system response to VAW in LMICs.

## Supplementary Information

**Additional file 1:** Details on client volume and services at OSCs in each setting.

## Data Availability

Not applicable to this article as no datasets were generated or analysed during the current study. A summary table synthesizing findings by the Health Systems Framework for Response to Violence Against Women is available from the corresponding author upon request.

## Abbreviations

BRAC: Building Resource Across Communities
GNI: Gross national income
HERA: Healthcare Responding to Violence and Abuse
HMIS: Health Management Information System
LIVES: Listen, Inquire about Needs and Concerns, Validate, Enhance Safety, and Support to Information and Services
LMIC: Low- and middle- income country
NGO: Nongovernmental Organization
NHSDP: Nongovernmental Organization Health Service Delivery Project
OCMC: One-Stop Crisis Management Center
OSC: One-stop center
UNFPA: The United Nations Population Fund
VAW: Violence against women
WHO: World Health Organization

## Footnotes

1 LIVES refers to immediate empathic care to be provided to survivors, with components consisting of Listen, Inquire about needs and concerns, Validate, Enhance safety, and Support to information and services [31].
