# Supplementary material for: Mapping the health systems response to violence against women: key learnings from five LMIC settings (2015–2020)
Source: BMC Womens Health. 2021 Oct 10;21:360. doi: 10.1186/s12905-021-01499-8 (PMC8504083; doi:10.1186/s12905-021-01499-8)
Supplement: Supplementary file 1 — Additional file 1: Details on client volume and services at OSCs in each setting. [file 12905_2021_1499_MOESM1_ESM.docx]

Manuscript Title: Mapping the health systems response to violence against women: Key learnings from five LMIC settings (2015-2020)

Author List: Dr. Shegufta Shefa Sikder, CARE USA, 151 Ellis St NE, Atlanta GA, 30303, USA, [shefa.sikder@care.org](mailto:shefa.sikder@care.org); Dr. Rakhi Ghoshal, CARE India, No.14, Patliputra Colony, Bihar 800013, India, [rakhi.ghoshal@gmail.com](mailto:rakhi.ghoshal@gmail.com); Dr. Padma Bhate-Deosthali, Independent Consultant, India, [padma.deosthali@gmail.com](mailto:padma.deosthali@gmail.com); Dr. Chandni Jaishwal, Rollins School of Public Health, Emory University, 1518 Clifton Rd, NE Atlanta, GA, 30322, USA, [cjaishw@emory.edu](mailto:cjaishw@emory.edu); Corresponding author: Dr. Nobhojit Roy MD, MPH, PhD. Department of Global Public Health. Karolinska Institutet, SE-171 77 Stockholm, Sweden [nobhojit.roy@ki.se](mailto:nobhojit.roy@ki.se)

**Additional File 1. Available Details on One Stop Centres within Selected Low- and Middle-Income Countries**

| **Country** | **Volume of Clients** | **Causes of Violence** | **Who Clients Came with** | **Services Received** |
| --- | --- | --- | --- | --- |
| Bangladesh | An impact report of the second phase of Multi Sectoral Program on VAW (2008-2011) found that 19,286 females availed care from 7 OSCs between 2001 and April 2014 [35]. | Physical violence (71%), rape or sexual violence (19%), burns (7%), economic violence (3%) [34]. | Police (43%)  Neighbors or friends (17%)  Relatives (34%)  Women’s organizations (4%)  On their own (6%) | 85% of women expressed satisfaction with the services received at the OSC.  76% reported receiving a health checkup (UGA, DNA, blood test etc), 23% received nutrition support, 11% mentioned free burn medication, 3% mentioned other health treatments. The average stay was 5.7 days. |
| Brazil | Not Available | Not Available | Not Available | Not Available |
| Nepal | In a 2020 scale up report of one stop centers, the total annual number of clients increased from 187 in 2011/12 (based on 7 reporting facilities) to 6,992 in 2018/19 (based on 45 reporting facilities) [27]. | From 2018/19:  Rape and sexual assault (38%) Physical assault (34%) Emotional abuse (14%) Economic abuse (11%) Child marriage (1%) [27] | Not Available | A self-assessment scorecard completed by 50 OSCs in March 2020 showed the highest score on coordination and collaboration within the hospital, and the lowest score on preparation of a joint action plan with local GBV service providers including police and local government [27]. |
| Rwanda | Not available | Not available | Not available | Between 2016 and 2018, analysis from application of quality assurance standards showed a 20% increase in performance among 12 OSCs, particularly in empathetic counselling, referral and follow-up of survivors.  Gaps remained in turnover of trained staff, staff shortages, and lack of supportive hospital leadership to address VAW issues [28]. |
| Sri Lanka | The number of clients seeking care grew from 447 in 2011 to 7,463 in 2017. | Of the 42,843 clients recorded from 2011 to July 2020:  physical assault cases (66%)  sexual assault cases (33%) | Not Available | In 2017, staff at OSCs (locally called Mithuru Piyasa centers) reported conducting follow up consultations with 63% of clients [41], |
